# The dental infections in patients undergoing preoperative dental examination before surgical treatment of saccular intracranial aneurysm

**DOI:** 10.1186/s13104-018-3704-z

**Published:** 2018-08-20

**Authors:** Mikko J. Pyysalo, Liisa M. Pyysalo, Jenni Hiltunen, Jorma Järnstedt, Mika Helminen, Pekka J. Karhunen, Tanja Pessi

**Affiliations:** 1 Department of Oral and Maxillofacial Diseases, Tampere University Hospital, P O Box 2000, 33521 Tampere, Finland; 2 Oral Health Services, City of Tampere, Tampere, Finland; 3 Department of Neurosurgery, Tampere University Hospital, Tampere, Finland; 4 Faculty of Medicine and Life Sciences, University of Tampere and Fimlab Laboratories Ltd, Pirkanmaa Hospital District, Tampere, Finland; 5 Medical Imaging Centre, Department of Radiology, Tampere University Hospital, Tampere, Finland; 6 Science Centre, Pirkanmaa Hospital District, Tampere, Finland; 7 School of Health Sciences, University of Tampere, Tampere, Finland; 8 Department of Clinical Pathology and Forensic Medicine, University of Kuopio, Kuopio, Finland

**Keywords:** Intracranial aneurysm, Oral health, Inflammation, Periodontitis, Bacteria, qPCR, Bacterial DNA, Fusobacteria

## Abstract

**Objective:**

Dental bacterial DNA and bacterial-driven inflammation markers have previously been detected in intracranial aneurysm tissue samples. This study aimed (i) to assess the possible presence of dental infectious foci, (ii) and the possible association between typical odontogenic bacteria and clinical dental findings in patients undergoing pre-operative dental examination before surgical treatment of saccular intracranial aneurysm. Ninety patients with an intracranial aneurysm were recruited to the study, and the patients’ teeth were routinely investigated. Clinical data and bacterial samples from the gingival pockets were collected from a subpopulation of 60 patients. Five typical dental pathogens and total bacteria amounts were measured from gingival samples using real-time quantitative PCR.

**Results:**

The amounts of total bacterial and *Fusobacterium nucleatum* DNA were significantly higher in the patients with ≥ 6 mm gingival pockets than patients without them (p < 0.01 and p < 0.01, respectively). A total of 43% of patients with an aneurysm had gingival pockets of 6 mm or deeper. Dental infectious foci are fairly common in the Finnish population, with the prevalence of severe periodontitis being around 20%. The frequency of chronic dental infections, especially periodontitis seems to be higher in patients with intracranial aneurysm.

**Electronic supplementary material:**

The online version of this article (10.1186/s13104-018-3704-z) contains supplementary material, which is available to authorized users.

## Introduction

The most common chronic oral bacterial disease is periodontitis, and the incidence of severe periodontitis is approximately 20% in the Finnish population [1]. Infection in the gingival pocket leads to pocket deepening, thus the depth of gingival pockets is used as an indicator of the severity of periodontal disease [2]. Gingival pockets deeper than 6 mm are considered a sign of severe periodontitis. Another common dental infection type is endodontic infection, which eventually leads to a radiologically visible periapical lesion. Chronic periodontitis increases the amount of the systemic inflammatory mediators C-reactive protein (CRP) and interleukin-6 (IL-6), as well as total cholesterol and low-density lipoprotein (LDL) cholesterol levels [3]. Oral infections are suggested to play an important role in the pathogenesis of cardiovascular diseases, including cerebrovascular diseases, although the direct causality has not been shown reliably [4–7].

Periodontal pathogens such as *Treponema denticola* and *Porphyromonas gingivalis* have been identified in the cerebrospinal fluid (CSF) and neuronal ganglia [8, 9]. It has been shown that *Porphyromonas gingivalis* has the ability to invade brain tissue through the blood–brain barrier leading to neuronal injury via complement C3 activation [8]. Periodontal pathogens have been suggested to be involved in the rupturing process of intracranial aneurysms. In our previous study, we detected bacterial DNA of oral origin in 58% of the intracranial aneurysm tissue samples and observed highly intensive staining of the inflammatory/bacterial receptors CD14 and toll-like receptor-2 in ruptured aneurysms [10, 11]. The link between dental infections and cerebral diseases has also been suggested by others [12–14], but to the best of our knowledge, there are no other studies assessing the association between the dental infectious burden and the gingival bacterial profiles of patients with subarachnoid haemorrhage.

The aim of this study was to assess the presence of dental infectious foci and to investigate the association between typical periodontal/endodontic pathogens and clinical dental parameters in patients undergoing preoperative dental examination before surgical treatment of saccular intracranial aneurysm.

## Main text

### Materials and methods

Ninety patients undergoing preoperative dental examination due to planned surgical treatment of an intracranial aneurysm were recruited to the study between September 2012 and December 2014. Of the patients, 30 presented with a ruptured aneurysm and 60 with an unruptured aneurysm. The inclusion criteria were having a saccular aneurysm, being aged between 18 and 99 years, having the ability to give informed written consent, and having a clinical condition allowing safe transportation to the radiological department in the same building.

The patients’ teeth were investigated during the acute hospitalization period of aneurysm treatment by an experienced oral and maxillofacial surgeon (MP) and a study nurse. A routine clinical oral investigation was performed. Both panoramic tomography (Scanora 3Dx, Tuusula, Finland; 73 kV, 10 mA, 15 s) and dental cone beam tomography (Scanora 3Dx, Tuusula, Finland; 90 kV, 10 mA, 2.4 s, voxel size 0.2 mm) were performed for all the patients. Smoking habits were recorded. Periodontal probing was carried out using a standard WHO periodontal probe (LM 8-550B Si, LM-instruments Ltd, Parainen, Finland) with about 20 g force. Gingival pockets were measured from 6 sites of each tooth and teeth, which had one or more (out of the six sites measured) ≥ 4 mm gingival pocket, were recorded as infection foci. Radiological images were examined by an experienced dental radiologist (JJ). The presence of furcation lesions, vertical pockets and dentine carious lesions were assessed from the panoramic tomography. Horizontal bone loss was measured as millimeters from the cementoenamel junction and considered positive if it was > 2 mm. The amount and location of periapical lesions were assessed using the dental cone beam tomography.

Samples for bacterial DNA analyses were taken from the deepest gingival pocket crevicular fluid of the subpopulation of the 60 patients with an unruptured aneurysm using a sterile blotting paper pin (Pearl Dent Co, Ho Chi Minh City, Vietnam).

For the quantitative real-time PCR, the samples were equalized according to the measurement of NanoDrop (NanoDrop Products Ltd, Wilmington, USA). The DNA from five selected typical periodontal and endodontic bacteria (*Streptococcus* sp. mainly *Str. mitis* group bacteria, *Prevotella intermedia*, *Fusobacterium nucleatum*, *Aggregatibacter actinomycetemcomitans*, *Porphyromonas gingivalis*) and the total amount of bacterial DNA (universal bacterial measurement) were measured as previously described [15] by real-time quantitative PCR (RT-qPCR) using the AbiPrism 7900 HT Sequence Detection System (Applied Biosystems Ltd, California, USA). Shortly, for each real-time PCR, 20 μl of a mixture containing 1 μl of DNA extract, 1× TaqMan Universal Environmental PCR Master Mix (Applied Biosystems, Foster City, Calif., USA), 900 nM (each) sense and antisense primer, and 200 nM TaqMan probe were placed in each well of a 384-well plate. Amplification and detection were performed using the ABI PRISM 7900 sequence detection system (Applied Biosystems). PCR was performed twice as duplicates of each sample. In uncertain cases, analyses were repeated. Negative and positive controls were included with each batch of samples being analysed. The positive control comprised a standard PCR reaction mixture containing 5 ng of each reference bacteria listed below and the negative control contained sterile water instead of the sample. Reference bacteria from the ATCC collection (*Streptococcus mitis* ATCC 49456, *Prevotella intermedia* ATCC 25611, *Fusobacterium nucleatum* subsp. *nucleatum* ATCC 25586, *Aggregatibacter actinomycetemcomitans* ATCC 700685, *Porphyromonas gingivalis* ATCC 33277, *E. coli* strain ATCC 25922) were used to determine standard curves. From the standard curves, the absolute amount of bacterial DNA was calculated using the formula: Ct = − slope * log(x) + constant. The cut-off values, i.e. detection limits, were 40 cycles for all candidate bacteria, except for the *Streptococcus* sp. (mainly the *Str. mitis*) group (37 cycles) and the universal bacterial measurement (34 cycles).

Fisher’s exact test were used to compare the prevalences of periapical lesions and gingival pockets ≥ 6 mm between smokers and non-smokers.

The absolute amounts of measured bacterial DNA were skewed, thus logarithmic values were used in the Mann–Whitney test to assess the differences in the amounts of bacterial DNA in the samples between the patients with and without ≥ 6 mm gingival pockets.

Statistical analyses described above were performed with R software [R Core Team (2013). R: A language and environment for statistical computing. R Foundation for Statistical Computing, Vienna, Austria. URL: http://www.R-project.org/].

### Results and discussion

All the clinical results are presented in Table 1. Of the 89 aneurysm patients, 56 were females and 33 were males. The mean age of the study group was 52.6 years (median = 55.5, SD = 10.6). None of the patients had diabetes.

**Table 1:** Patient characteristics and the results of clinical and radiological assessments of infection parameters of the aneurysm patients

	Aneurysm patients
	n = 89 (%)
Sex	
Male	37
Female	63
Smoking	
Yes	52
4–5 mm pockets	
Yes	75
≥ 6 mm pockets	
Yes	43
Furcation lesions	
Yes	18
Periapical lesions	
Yes	39
Dentine caries lesions	
Yes	22
Tooth brushing	
Twice a day	61
Once a day	28
Not every day	10
Bone loss	
Yes	66
Vertical pockets	
Yes	22

Our study shows that altogether 75% of the patients had one or more 4–5 mm-deep gingival pocket. The number of pockets ranged from 0 to 15 (out of positive findings: median = 3, mean = 3.22). Gingival pockets ≥ 6 mm were seen in 43% of the patients. The number of such pockets ranged from 0 to 14 (out of positive findings: median = 2, mean = 3.00). Dental infectious foci are not uncommon in the Finnish population. In Finnish population based “Health 2000” study, the prevalence of deep gingival pockets (6 mm or deeper) was reported to be much lower, i.e. 21% [1]. Periapical lesions were detected using radiographs, as described in materials and methods, in 38% of the patients, the amount ranging from 0 to 6 (out of positive findings: median = 1, mean = 1.68). In general population [1], the amount of periapical lesions were similar. However, 53% of male patients with an aneurysm had periapical lesions whereas in population based study the frequency of the lesions in males was 31%. Our results were not biased by smoking (data not shown).

In terms of clinical examination methodology, the results between this study and population based Health 2000 study can be considered comparable. We used similar WHO standard periodontal probe with a standard 20 g force (LM 8-550B Si, LM-instruments Ltd, Parainen, Finland) that in the control survey. In Health 2000 survey the clinical examinations were performed in a dental appointment room. In our study, the examination was performed in a neurosurgical ward or in an operation theatre, depending on a clinical condition of the patient. We used additional light (BFV Vistaview II, Louisville, Kentucky, USA) and 2.8×–3.2× magnifying loupes (Custom made by Surgical Acuity, Wisconsin, Middleton, USA) in every clinical examination to mimic dental appointment room conditions. In this study both dental cone beam tomography and panoramic tomography were performed for all the patients whereas in Health 2000 study only panoramic tomography was used. Dental cone beam tomography is considered more accurate imaging modality than panoramic tomography especially in upper molar palatal root area. Thus there may be certain bias in assessed periapical lesions in upper molar area when comparing these two studies.

**Fig. 1:**
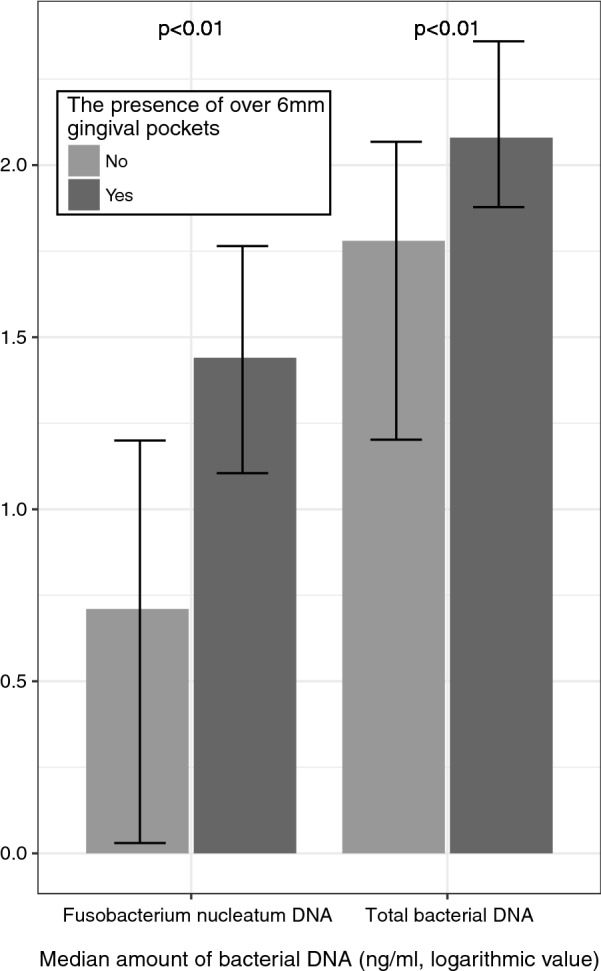
The median amount of total bacterial DNA and DNA from Fusobacterium nucleatum in patients with unruptured intracranial aneurysms with and without ≤ 6 mm gingival pockets

Amount of gingival bacterial DNA from the sub-cohort of 60 patients with an unruptured aneurysm showed that the total amounts of bacterial and *Fusobacterium nucleatum* DNA were significantly higher in the patients with ≥ 6 mm gingival pockets than patients without them (Mann–Whitney-test; *p *< 0.01 and *p *< 0.01, respectively; Fig. 1). No significant differences were observed among the other bacterial DNA measured. *Fusobacterium nucleatum* is a common periodontal pathogen that plays a key role in pathogenic subgingival biofilm formation by bridging together the early colonizers, like streptococci and actinomyces, and the late colonizers [16]. Subgingival biofilm within active periodontal lesions may further initiate destructive periodontitis even until bone loss [17]. Adhesion molecules of *F. nucleatum* [18], as well as the invasive properties of *F. nucleatum* [19], may allow bacteria to enter the bloodstream, migrate, and cause infections elsewhere in the body [20]. In our previous study, an increased amount of bacterial DNA and the presence of the same periodontal bacterial species, i.e. *Fusobacterium nucleatum*, along with streptococci was also detected in the aneurysm tissue samples [11], suggesting that fusobacteria may escape from oral infection foci and be involved in tissue inflammation within the cerebral wall.

### Conclusions

There are many studies on the general prevalence of periodontitis summarized by Kassebaum et al. [21], and there are a few studies concerning the possible association between chronic dental infection and stroke [22, 23], but to the best of our knowledge, there are no other studies on the association between these infections and subarachnoid haemorrhage. In the present study, we showed that deep periodontal inflammatory pockets and periapical lesions were more common in patients with a cerebral aneurysm than in the normal population. The results are not biased by smoking or diabetes. In addition, gingival pocket samples from aneurysm patients with periodontitis contained more bacterial DNA and *Fusobacterium nucleatum* DNA than non-inflamed gingivae: the same bacteria and increased bacterial amount were previously detected to be the most common finding in ruptured and non-ruptured cerebral aneurysm samples. Although the data in this study is cross-sectional and thus does not directly support a cause-effect relationship, these findings together suggest that there may be an association between chronic dental infections, especially periodontitis, and saccular intracranial aneurysms. However, these results need verification in a larger cohort.

## Limitations

We were not able to collect the gingival samples from the controls to perform case–control study. However, the prevalence of dental infections as well as amount of the typical odontogenic taxa in gingival pockets are well reported in many studies. Instead collecting own controls we used data from population-based study, the “Health 2000”. The survey consists of 6335 clinically examined patients and 6114 panoramic tomographies, representing well the normal Finnish population. The sample size of this study was limited, thus not allowing us to divide the patients into reliable representative subgroups. The patients in this study are mostly different from our earlier studies [10, 11]. We were not able to determine in this study if high fusobacteria levels in gingival pockets correlate with bacterial findings per se in the aneurysm. Moreover, there is no consensus on which oral bacterial taxa are the most significant in the oral cavity and relevant to measure in the gingival pockets of patients with intracranial aneurysms. Five candidates and the total bacterial measurement were selected here, since the selected oral bacterial taxa are those most frequently associated in infection foci within both the oral cavity and the vascular wall [10, 11, 24–27].

## Additional files

**Additional file 1.** Demographic and clinical findings of all patients.

**Additional file 2.** Demographic, clinical and bacterial DNA findings of the patients with unruptured aneurysm.

**Additional file 3.** Measuring the depth of a gingival pocket.

**Additional file 4.** Sampling gingival crevicular fluid with a paper pin.

## Abbreviations

DNA: deoxyribonucleic acid
PCR: polymerase chain reaction
mm: millimetre
CRP: C-reactive protein
IL-6: interleukin-6
LDL: low-density lipoprotein
CSF: cerebrospinal fluid
CD14: cluster of differentiation 14
kV: kilovolt
mA: milliampere
g: gram
μl: microliter
nm: nanometre
ng: nanogram
