# Supplementary figures and images for: The dental infections in patients undergoing preoperative dental examination before surgical treatment of saccular intracranial aneurysm
Source: BMC Res Notes. 2018 Aug 20;11:600. doi: 10.1186/s13104-018-3704-z (PMC6102815; doi:10.1186/s13104-018-3704-z)

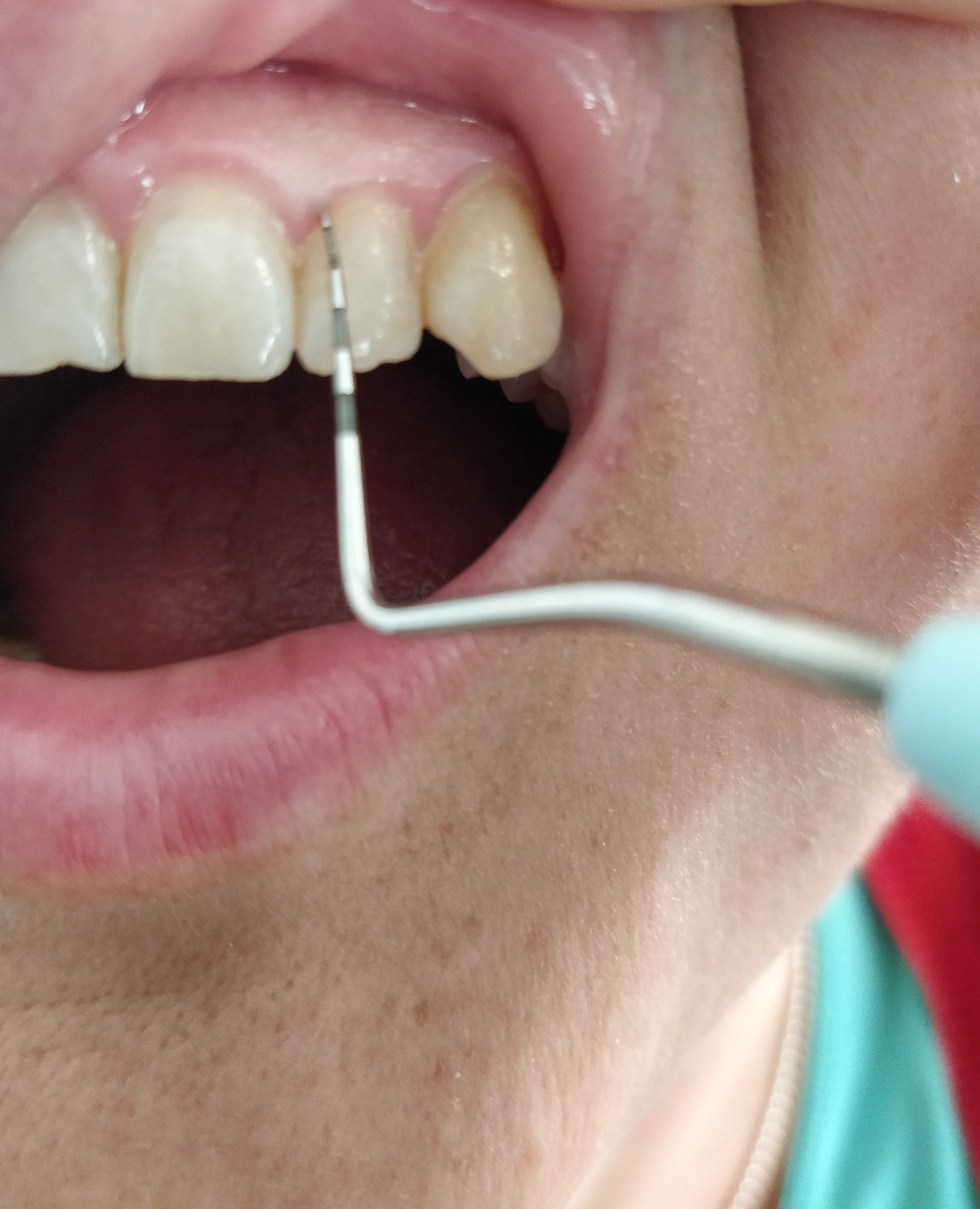

Supplement: Supplementary file 3 — Additional file 3. Measuring the depth of a gingival pocket. [file 13104_2018_3704_MOESM3_ESM.jpg]

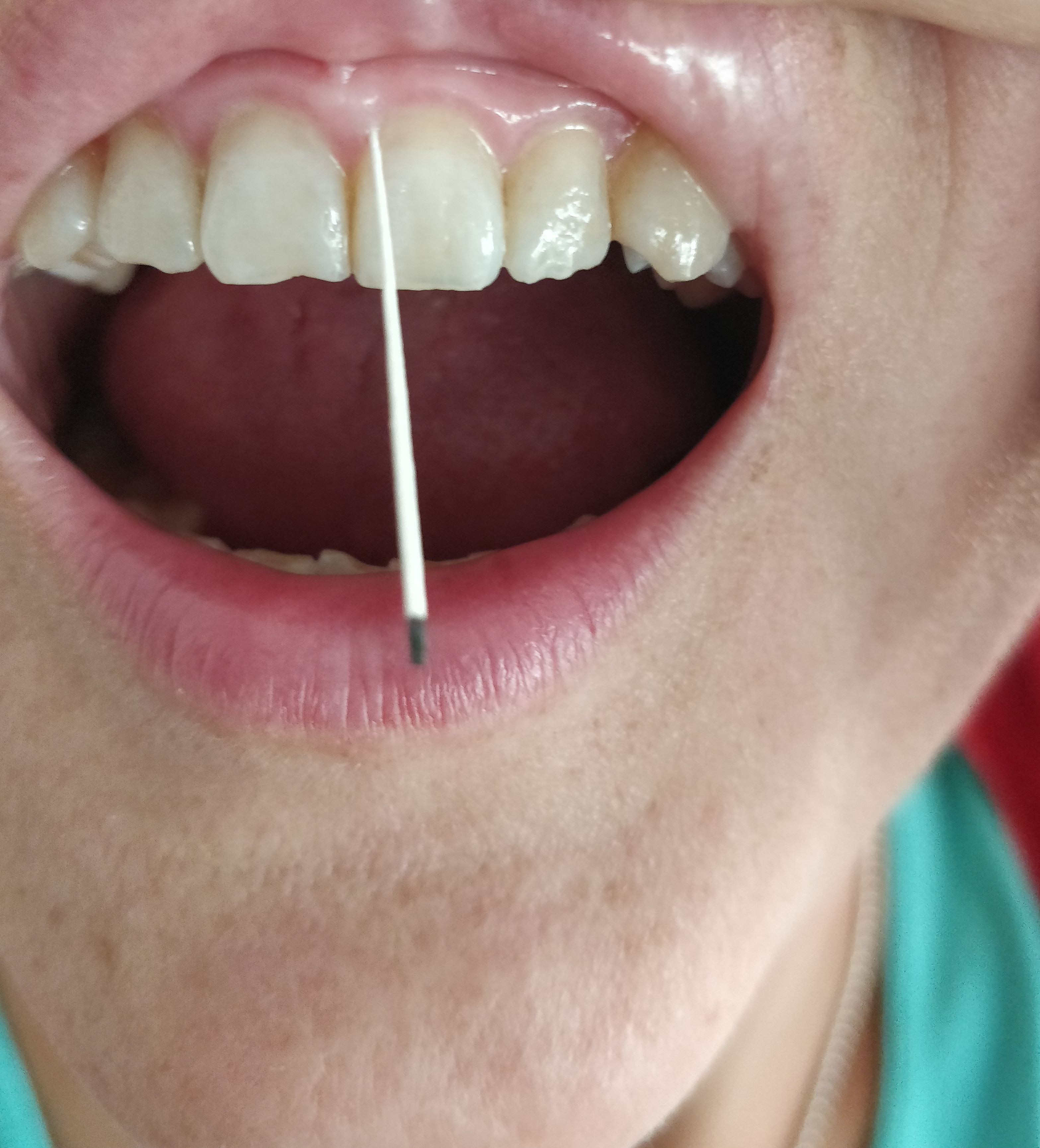

Supplement: Supplementary file 4 — Additional file 4. Sampling gingival crevicular fluid with a paper pin. [file 13104_2018_3704_MOESM4_ESM.jpg]
